# *Ex vivo* Quantitative Proteomic Analysis of Serotonin Transporter Interactome: Network Impact of the SERT Ala56 Coding Variant

**DOI:** 10.3389/fnmol.2020.00089

**Published:** 2020-06-08

**Authors:** Meagan A. Quinlan, Matthew J. Robson, Ran Ye, Kristie L. Rose, Kevin L. Schey, Randy D. Blakely

**Affiliations:** ^1^Department of Psychiatry and Behavioral Sciences, University of Washington, Seattle, WA, United States; ^2^Department of Pharmacology, Vanderbilt University, Nashville, TN, United States; ^3^Department of Biomedical Science, Charles E. Schmidt College of Medicine, Florida Atlantic University, Jupiter, FL, United States; ^4^Division of Pharmaceutical Sciences, James L. Winkle College of Pharmacy, University of Cincinnati, Cincinnati, OH, United States; ^5^Department of Biochemistry, Vanderbilt University, Nashville, TN, United States; ^6^Brain Institute, Florida Atlantic University, Jupiter, FL, United States

**Keywords:** serotonin transporter, SERT interacting proteins, autism spectrum disorder (ASD), quantitative proteomic analysis, SERT regulation

## Abstract

Altered serotonin (5-HT) signaling is associated with multiple brain disorders, including major depressive disorder (MDD), obsessive-compulsive disorder (OCD), and autism spectrum disorder (ASD). The presynaptic, high-affinity 5-HT transporter (SERT) tightly regulates 5-HT clearance after release from serotonergic neurons in the brain and enteric nervous systems, among other sites. Accumulating evidence suggests that SERT is dynamically regulated in distinct activity states as a result of environmental and intracellular stimuli, with regulation perturbed by disease-associated coding variants. Our lab identified a rare, hypermorphic SERT coding substitution, Gly56Ala, in subjects with ASD, finding that the Ala56 variant stabilizes a high-affinity outward-facing conformation (SERT^∗^) that leads to elevated 5-HT uptake *in vitro* and *in vivo*. Hyperactive SERT Ala56 appears to preclude further activity enhancements by p38α mitogen-activated protein kinase (MAPK) and can be normalized by pharmacological p38α MAPK inhibition, consistent with SERT Ala56 mimicking, constitutively, a high-activity conformation entered into transiently by p38α MAPK activation. We hypothesize that changes in SERT-interacting proteins (SIPs) support the shift of SERT into the SERT^∗^ state which may be captured by comparing the composition of SERT Ala56 protein complexes with those of wildtype (WT) SERT, defining specific interactions through comparisons of protein complexes recovered using preparations from SERT^–/–^ (knockout; KO) mice. Using quantitative proteomic-based approaches, we identify a total of 459 SIPs, that demonstrate both SERT specificity and sensitivity to the Gly56Ala substitution, with a striking bias being a loss of SIP interactions with SERT Ala56 compared to WT SERT. Among this group are previously validated SIPs, such as flotillin-1 (FLOT1) and protein phosphatase 2A (PP2A), whose functions are believed to contribute to SERT microdomain localization and regulation. Interestingly, our studies nominate a number of novel SIPs implicated in ASD, including fragile X mental retardation 1 protein (FMR1) and SH3 and multiple ankyrin repeat domains protein 3 (SHANK3), of potential relevance to long-standing evidence of serotonergic contributions to ASD. Further investigation of these SIPs, and the broader networks they engage, may afford a greater understanding of ASD as well as other brain and peripheral disorders associated with perturbed 5-HT signaling.

## Introduction

Disruptions in serotonergic signaling have been implicated in a wide array of neurological disorders, including major depressive disorder (MDD) (Plaznik et al., 1989; Meltzer, 1990), autism spectrum disorder (ASD) (Cook et al., 1997; Muller et al., 2016) and obsessive–compulsive disorder (OCD) (Sinopoli et al., 2017). The primary regulator of the termination of 5-HT signaling is the presynaptic, high-affinity 5-HT transporter (SERT), which rapidly clears 5-HT from the extracellular space. Evidence suggests that SERT responds to internal and external stimuli to modulate 5-HT uptake activity, ultimately regulating the availability, in space and time, of extracellular 5-HT. We and others have shown that whereas protein kinase C (PKC) activation triggers rapid reductions in both SERT activity (Jayanthi et al., 2005) and surface density (Qian et al., 1997), which can be attenuated by ongoing transport of 5-HT (Ramamoorthy and Blakely, 1999), protein kinase G (PKG) and p38α mitogen-activated kinase (MAPK) pathways rapidly elevate SERT-mediated 5-HT transport, with both catalytic activation and increased surface trafficking reported (Miller and Hoffman, 1994; Zhu et al., 2004, 2005; Samuvel et al., 2005).

Single particle labeling studies reveal that regulation of SERT by PKG/p38α MAPK pathways leads to changes in SERT surface mobility mobility, with functional and mobility effects mimicked by treatment with the actin cytoskeleton destabilizer cytochalasin D (Chang et al., 2012), suggesting surface transporter anchoring by one or more actin-associated proteins. Interestingly, stimulation of phosphatase and kinase-linked pathways that modulate SERT activity, such as protein phosphatase 2A (PP2A), nitric oxide synthase 1 (NOS1), p38α MAPK and PKG have also been shown to modulate SERT interacting proteins (SIPs) (Bauman et al., 2000; Samuvel et al., 2005; Chanrion et al., 2007; Zhu et al., 2011), and other SIPS have been identified and found to impact SERT protein trafficking, activity, stability (Carneiro and Blakely, 2006; Müller et al., 2006; Mouri et al., 2012; Reisinger et al., 2018). Pharmacological activation of intracellular signaling pathways can be mimicked by stimulation of cell surface receptors in transfected and natively expressing preparations, including the A3 adenosine receptor (A3AR) (Miller and Hoffman, 1994; Zhu et al., 2004, 2007), kappa opiate receptors (Sundaramurthy et al., 2017), integrin b3 (ITGB3) receptor (Carneiro et al., 2008), and interleukin 1 receptor (IL-1R) (Zhu et al., 2010), with A3ARs and ITGB3 receptors reported to be SIPs (Carneiro et al., 2008; Zhu et al., 2011). Thus, it is hypothesized SERT activity is defined and regulated by distinct SIP complexes that can include receptors as well as intracellular signaling machinery. Clearly defining the SIPs, particularly in relation to transporter regulation and the impact of mutations, may be useful in identifying novel drug targets to manage 5-HT associated disorders such as depression. These SIPs may also provide a path to indirect targeting of SERT, of interest as effective dosages of SERT-targeted, 5-HT selective reuptake inhibitors (SSRIs) require nearly total occupancy of the transporter, precluding its normal and important contribution to 5-HT signaling. More subtle therapeutics that alter or overcome the actions of SIPs may allow for a restoration of normal, physiological 5-HT signaling with the appropriate control mechanisms intact.

Identifying SIPs, and linking these to transport function and disease, is technically challenging, as SERT does not exist in a static state with a constitutively defined set of proteins (Steiner et al., 2008). We hypothesized that use of an *in vivo* expressed SERT mutant, stabilized in a disease-associated conformation, could elucidate both regulatory SIPs as well as SIPs contributing to pathophysiology. Thus, whereas SIPs might interact only transiently with WT SERT, mutant-induced conformations might stabilize or limit associations to a degree that would enhance identification. In this regard, the Blakely lab identified the gain-of-function, autism-associated coding variant, SERT Ala56 (Prasad et al., 2005, 2009; Sutcliffe et al., 2005), which more recent studies indicates is stabilized in a high-affinity state associated with increased activity (SERT^∗^) (Quinlan et al., 2019). Importantly, the availability of a SERT Ala56 knock-in mouse (Veenstra-VanderWeele et al., 2009, 2012) provides a natively expressed pool of conformationally stabilized transporters from which proteins that prefer (or not) the SERT^∗^ state could be identified. Below, we describe our efforts in utilizing synaptic preparations from SERT Ala56 and wildtype (WT) littermates, as well as from SERT^–/–^ (knockout; KO) mice (Bengel et al., 1998) to validate SERT interaction specificity and to identify SERT regulatory complexes whose altered interactions may contribute to pathophysiology.

## Results

### Identification of SERT Complexes by Immunoprecipitation

In order to identify novel SIPs using an unbiased approach, we pursued a proteomic analysis using immunoisolation of SERT protein complexes from midbrain synaptosomes, prepared from WT, SERT Ala56, and SERT KO mice. We limited our studies to male mice since past studies of SERT Ala56 mice have been restricted to males (Veenstra-VanderWeele et al., 2012; Robson et al., 2018; Quinlan et al., 2019), primarily due to the 4:1 male bias found in ASD (Baio et al., 2018). However, future studies would clearly benefit from understanding the sex difference in SERT functional regulation, which may provide evidence to explain the male bias present in ASD. We selected the midbrain region to identify SERT complexes as the midbrain has the highest expression of CNS SERT and also contains SERT in both somatodendritic and axonal compartments (Ye et al., 2016). Differences in transporter proteins interactions in various brain regions have been previously been reported in our lab for both SERT (Ye et al., 2016) and dopamine transporter (DAT) (Gowrishankar et al., 2018). Mapping the SERT interactome and its perturbation by the SERT Ala56 variant outside the midbrain will likely lead to additional insights into the regulation of SERT trafficking and function.

**FIGURE 1:**
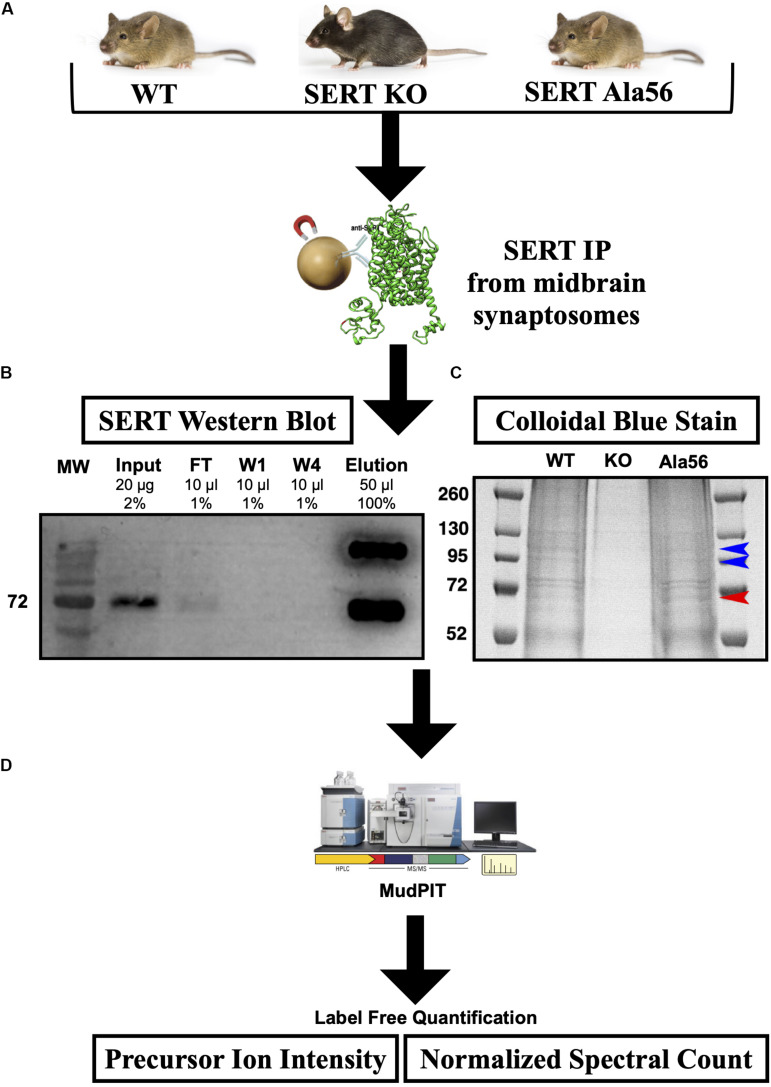
SERT co-immunoprecipitation followed by LC-MS/MS. **(A)** Midbrain synaptosomes from WT, SERT KO (negative control), and SERT Ala56 mice were affinity purified for SERT (*n* = 3). **(B)** Western blot confirms efficient SERT IP (MW = Molecular Weight; FT = Flow Through; W1 = Wash 1; W4 = Wash 4). **(C)** Colloidal-blue stain of proteins eluted from SERT IP confirms altered protein association profiles (red and blue arrow indicate increase and decrease, respectively, protein band intensities in SERT Ala56 compared to WT). **(D)** In-gel tryptic digestion was followed by an 8 step MudPIT. Eluting peptides were mass analyzed on an LTQ Orbitrap Velos (Thermo Scientific). Scaffold (version 4.7.3) was used to validate MS/MS based peptide and protein identifications.

SERT immunoprecipitations (IPs) were optimized, considering different detergents and conditions to afford maximal recovery of SERT proteins from midbrain synaptosomes as described in the “Materials and Methods” section. In order to maximize recovery of SERT, we pooled four midbrains per genotype and to account for variability across samples we performed three technical replicates (12 mice total used per genotype). SERT was IPed from midbrain synaptosomes utilizing a C-terminal SERT anti-serum #48 (epitope developed against amino acid 596-622 of rat SERT and detects both hSERT and rodent SERT; Qian et al., 1995) that was covalently cross-linked to magnetic protein A beads, as described in section “Materials and Methods” (Figure 1). Since the SERT Ala56 mutation is located within the N-terminus, we wanted to avoid using an antibody directed against the N-terminus, as the mutation could directly affect antibody recognition and result in false conclusions as to mutant impact on SIP interactions. Western blot studies indeed revealed that Ab48 immunoprecipitated equal quantities of synaptosomal SERT from WT and SERT Ala56 mice (data not shown).

SIP complexes were identified by using Multi-dimensional Protein Identification Technology (MudPIT) (Wolters et al., 2001), an application that allows for increased detection of peptides from LC-MS/MS. The resulting peptides from all samples matched to a total of 1050 proteins. To quantify SIPs in the different genotypes, two label-free quantifying techniques were employed, precursor ion intensity and normalized spectral counts. To eliminate non-specific proteins, proteins that were identified in samples from SERT KO mice with a 1.5-fold enrichment in SERT KO compared to WT or SERT Ala56 samples, by either precursor ion intensity or normalized spectral counts, were removed. This narrowed the list from 1050 proteins identified in this experiment to 459 specific SIPs (Supplementary Table 1). Importantly, there was no difference in SERT levels estimated between WT and SERT Ala56 samples by either ion intensity or normalized spectral count quantitation, indicating the utility of our methods for identifying and quantifying proteins in our immunocomplexes. These findings also match our previously published data showing no difference in SERT expression between SERT Ala56 and WT littermates when assessed by Western blot analysis (Veenstra-VanderWeele et al., 2012; Robson et al., 2018). Of note, two C-terminal peptides for SERT were identified in the SERT KO samples, which may be due to the fact that SERT KO mice produce a truncated SERT, that has been shown to remain somatodendritically and does not transport to the membrane, remaining mostly in the endoplasmic reticulum (Ravary et al., 2001). While this may have led to some C-terminally interacting proteins being dismissed in our analysis as specific, we believe that this missing contribution represents a small fraction of the peptides recovered from our WT and SERT Ala56 samples it should not drastically affect our results.

**FIGURE 2:**
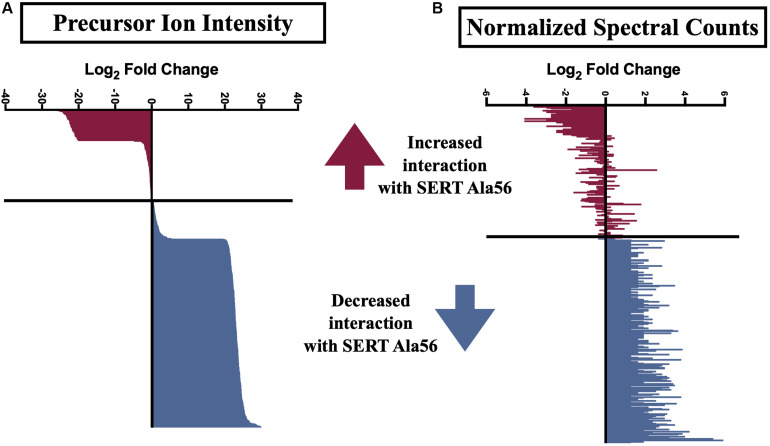
Log_2_ fold change of SIPs between SERT Ala56 and WT SERT. Log_2_ fold change of **(A)** precursor ion intensity and **(B)** normalized spectral counts. Proteins in red indicated proteins increased interaction with SERT Ala56 compared to WT and blue labeled proteins show decreased association with SERT Ala56 compared to WT SERT based on precursor ion intensity.

Several previously described SIPs were identified in our immunoisolates (noted by ^∗^ in Supplementary Table 1), as well as proteins found in a separate proteomic analysis from another lab (noted by ^∗∗^ in Supplementary Table 1) (Haase et al., 2017), further supporting the utility of our methods. In order to uncover SIPs that differentially interact with WT SERT compared to SERT Ala56, the log_2_ fold change in SIP levels from the WT/Ala56 ratio was calculated for both normalized spectral counts and precursor ion intensity (log_2_ fold change WT/SERT Ala56 of <−0.5 indicates increased interaction with SERT Ala56, log_2_ fold change WT/SERT Ala56 of >0.5 designates decreased interaction with SERT Ala56 and log_2_ fold change WT/SERT Ala56 between −0.5 and 0.5 signifies no difference between genotype; Supplementary Table 1). Due to the exploratory nature of our effort, and the complementary aspects of these methods of quantification, we required differences to be present in only one of the two approaches to consider for further analyses. As presented in Figure 2, most proteins show overlap between normalized spectral count and precursor ion intensity in quantifying differential SIPs across conditions, with only a few exceptions. For example, the calcium/calmodulin-dependent protein kinase type II subunit beta (CaMKIIβ; *Camk2b*) by normalized spectral count analysis demonstrated a log_2_ fold-change of 2.57, indicating a decreased interaction with SERT Ala56, relative to WT, but by ion intensity, the log_2_ fold-change WT/SERT Ala56 was -0.02, indicating no differences between genotypes.

Interestingly, appreciably more proteins were nominated as SIPs with WT SERT (308 proteins; 67.10% of total proteins) than with SERT Ala56 (69 proteins; 15.03% of total proteins) based on the log_2_ fold change of precursor ion intensity (Chi-square test *P* < 0.01). To provide some clarity on the association of the proteins within this list of 459 proteins, and to account for the possibility that we recover different components of the same complex at different stages in their interactions, network and functional clustering analysis was performed. Specifically, we performed network analysis based on the log_2_ fold change of precursor ion intensity proteins with increased and decreased interaction with SERT Ala56 as well as with proteins that showed no difference in interaction between genotypes (82 proteins; 17.86% of total proteins), and all proteins combined (459 proteins).

### Network Analysis of SIPs With Increased Interaction With SERT Ala56

There were 69 proteins with increased interaction with SERT Ala56 compared to WT SERT as defined by a log_2_ ion intensity of WT/Ala56 of less than −0.5. Functional annotation clustering from DAVID bioinformatics resource identified enrichment in proteins assigned to the cytoskeleton (GO:0005856), GTP binding (GO:0005525) and cell–cell adherens junction (GO:0005913) (Table 1). The input of these proteins into the STRING program, with interactions defined by genomic context predictions, high-throughput lab experiments, co-expression, automated text mining, and previous knowledge in databases, found this network has significantly more interactions than expected for a random similar sized set of proteins (protein–protein enrichment, Fisher’s exact test *P* = 0.00827).

**TABLE 1:** DAVID functional analysis of SIPs increased with SERT Ala56.

Annotation cluster	Enrichment score	Protein #	Gene name	*p*-value
Cytoskeleton	3.05	12	Mark1, Mark2, Add3, Capza1, Cep131, Coro1c, Coro2b, Pitpnm2, Sept11, Sept5, Sept8, Spta1	2.2E-04
Cytoplasm	3.05	25	Agap1, Mark1, Mark2, Nckap1, Upf1, Add3, Akr1b8, Capza1, Cep131, Coro1c, Coro2b, Fxr1, Fmr1, Gstp1, Hspa5, Homer 3, Osbpl3, Pitpnm2, Sept11, Sept5, Sept8, Spats2l, Tdrkh, Ywhah	7.5E-04
Cell-Cell Adherens Junction	3.05	7	Mark2, Arglu1, Capza1, Dlg1, Eeflg, Hspa5, Puf60	4.7E-04

Three proteins that have been previously identified as SIPs were included in the list of proteins with increased interaction with SERT Ala56 versus WT SERT: PP2A regulatory subunit B (PP2A; *Ppp2r2c*) (Bauman et al., 2000), protein transport protein Sec23a (*Sec23a*) (Chanrion et al., 2007; Sucic et al., 2011), and 14-3-3η (*Ywhah*) (Haase et al., 2001).

### Network Analysis of SIPs With Decreased Interactions With SERT Ala56

Of the 459 proteins nominated as SIPs, 308 were shown to have decreased interactions with SERT Ala56 compared to WT SERT. Functional annotation clustering from the DAVID online resource tool identified enrichment of proteins in a network assigned to kinase activity (GO:0016301), specifically serine/threonine kinases (GO:0004674), and amphetamine addiction (KEGG pathway) and Arp2/3 protein complex (GO:0005885) (Table 2). The input of these proteins collectively into the STRING program found this network has significantly more interactions than expected (protein-protein enrichment, Fisher’s exact test *P* < 1.0e-16), potentially suggesting they connected at least in part by a biologically relevant network.

**TABLE 2:** DAVID functional analysis of SIPs decreased with SERT Ala56.

Annotation cluster	Enrichment score	Protein #	Gene name	*p*-value
Kinase Activity	5.41	28	Araf, Cdc42bpb, Mark3, Mark4, Tnik, Agk, Cask, Csn1d, Csnk1d, Csnk2a1, Cerk, Cdk18, Cdkl5, Dgkz, Dgkb, Ddr1, Fn3k, Gk, Gsk3b, Magi3, Mast1, Mapk3, Mapk8ip2, Map2k1, Map3k13, Pfkl, Phkg1, Prkg2, Pdk3	7.4E-06
Amphetamine Addiction	3.66	6	Gria1, Gria2, Gria3, Gria4, Crin2a, Ppp1ca	9.4E-03
Arp2/3 Protein Complex	3.62	5	Actr3, Arpc2, Arpc3, Arpc4, Arpc5l	1.5E-05
Serine/Threonine Protein Kinase	3.08	17	Araf, Cdc42bpb, Mark3, Mark4, Tnik, Cask, Csnk1d, Csnk2a1, Cdk18, Cdkl5, Gsk3b, Mast1, Mapk3, Map3k13, Phkg1, Prkg2	1.1E-04

The protein that demonstrates the greatest reduction in associations with SERT Ala56, based on precursor ion intensity, is flotillin-1 (FLOT1; *flot1*), which has been previously shown to interact with DAT (Cremona et al., 2011; Sorkina et al., 2012) and SERT (Reisinger et al., 2018). Flotillin has been identified as a risk gene for MDD (Zhong et al., 2019), though the wide distribution of the protein makes it difficult to attribute specific behavioral changes to simply a serotonergic contribution. With respect to DAT, our lab demonstrated that an ADHD-associated DAT variant (Arg615Cys) also shows decreased interaction with FLOT1 and this change coincided with altered DAT localization within membrane lipid rafts (Sakrikar et al., 2012). SERT has been found to localize to lipid raft compartments and to exhibit dynamic, regulated mobility within these domains (Magnani et al., 2004). Interestingly, FLOT1 interaction with DAT is also necessary for amphetamine-induced changes in DAT function (Sakrikar et al., 2012; Pizzo et al., 2013). Considering SERT Ala56 shows a blunted D-fenfluramine-induced 5-HT efflux (Quinlan et al., 2019), diminished SERT Ala56: FLOT1 interactions may contribute to the changes in transporter functional states displayed by SERT Ala56, a hypothesis that requires further investigation.

### Network Analysis of SIPs That Show Similar Interaction With Both WT SERT and SERT Ala56

In our proteomic analysis, 82 of the 459 proteins (17.86% of total proteins) identified showed no difference in interaction with WT SERT and SERT Ala56, though their absence in SERT KO samples supports a SERT specificity to their interactions. Functional annotation clustering of these proteins utilizing the online DAVID platform showed enrichment in proteins assigned to Src Homology 3 (SH3) domain contacting proteins (UniProt keyword), GTPase activation (UniProt keyword), ATP binding (GO: 0005524), PDZ domain binding (GO: 0030165), and kinase activity (GO:0016301) (Table 3). Analysis of the network by the online STRING platform showed significantly more interactions than expected (protein–protein enrichment, Fisher’s exact test *P* = 2.8e-08).

**TABLE 3:** DAVID functional analysis of SIPs of similar interaction with WT SERT and SERT Ala56.

Annotation cluster	Enrichment score	Protein #	Gene name	*p*-value
SH3 Domain	6.51	11	Arhgap26, Arhgap32, Shank3, Srgap3, Yes1, Dlg2, Dlg4, Macf1, Sorbs1, Tjp2, Tnk2	1.0E-08
GTPase Activation	4.81	8	Agap2, Arhgap21. Arhgap23, Arhgap26, Arhgap32,Srgap3, Myo9a,Sipa1l1	3.9E-06
ATP Binding	4	19	Actr2, Abcd3, Atp9a, Agap2, Bmp2k, Yes1, Adck1, Acsl6, Camk2b, Csnk1a1, Csnk1d, Gak, Dgke, Mthfd1, Myo9a, Myo18a, Pcx, Stk39, Tnk2	1.1E-04
Guanylate Kinase/PDZ Domain Binding	3.16	4	Dlg2, Dlg4, Magi2, Tjp2	1.1E-04
Kinase Activity	2.28	11	Bmp2k, Yes1, Adck1, Camk2b, Csn1a1, Csnk1e, Gak, Dgke, Prkar2a, Stk39,Tnk2	9.0E-04

### Network Analysis of All Identified SIPs

**FIGURE 3:**
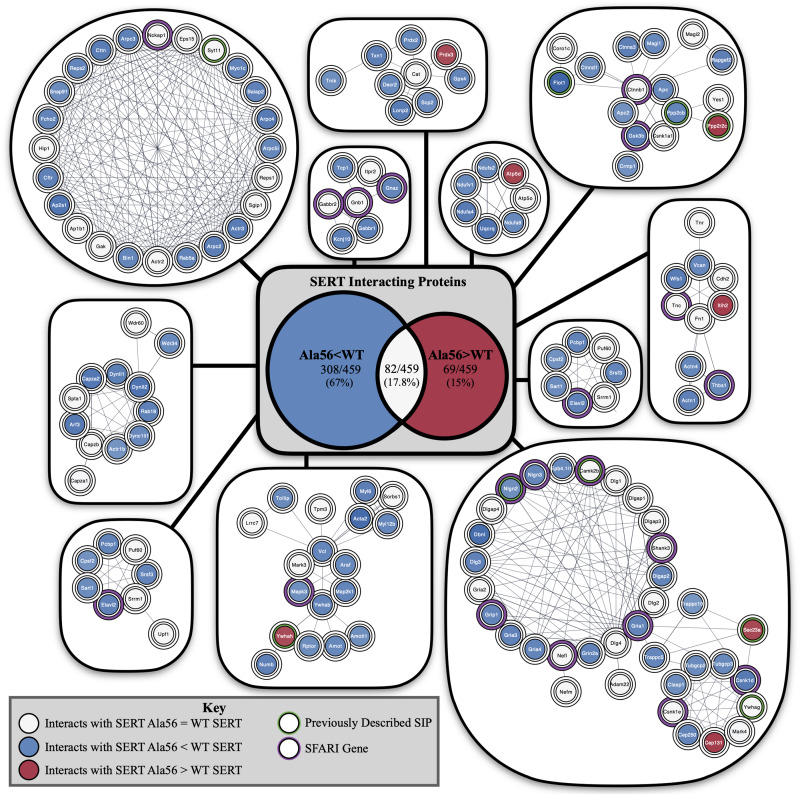
STRING network analysis of all interacting proteins. Network of protein–protein interactions from the Cytoscape Omics Visualizer plug-in with STRING (confidence score cut-off of 0.9) followed by application of the MCL algorithm using clusterMaker2 app, with a minimum size of 7 proteins per cluster. Highlighted proteins previously identified to interact with SERT with a green outline and identified SFARI genes with a purple outline. Center shows Venn diagram of proteins with decreased interactions with SERT Ala56 compared to WT SERT (blue), number of proteins with increased interactions with SERT Ala56 compared to WT SERT (red) and number of proteins with similar interaction with both SERT Ala56 and WT SERT (light gray).

Functional annotation clustering of all the proteins identified in this study utilizing the online DAVID platform showed enrichment in proteins assigned to SH3 domain-containing proteins (UniProt keyword), PDZ domain (UniProt sequence feature), kinase activity (GO:0016301) and receptor clustering (GO:0043113) (Table 4). Input of these proteins into the STRING program found this network has significantly more interactions than expected (protein–protein enrichment, Fisher’s exact test *P* < 1.0e-16). To visualize protein–protein networks based on STRING database of all proteins identified, the Cytoscape (Shannon et al., 2003) Omics Visualizer plug-in (employs Cytoscape StringApp, Doncheva et al., 2019) was utilized followed by application of the Markov Clustering (MCL) algorithm (Enright et al., 2002; Morris et al., 2011) to group interactions based on protein families (Figure 3).

**TABLE 4:** DAVID functional analysis of all identified SIPs.

Annotation cluster	Enrichment score	Protein #	Gene name	*p*-value
SH3 Domain	9.36	25	Caskin1, Arhgap26, Arhgap32, Arhgap42, Arhgef7, Shank3, Srgap3, Yes1, Baiap2, Bin1, Cask, Cttn, Dlg1, Dlg2, Dlg3, Dlg4, Dbnl, Macf1, Mapk8ip2, Myo1f, Sorbs1, Sorbs2, Spta1, Tjp2, Tnk2	6.6E-12
PDZ Domain	8.83	11	Dlg3, Dlg2, Dlg3, Dlg4, Gripl, Magi1, Magi2, Magi3, Pard3, Ptpnl3, Tjp2	1.2E-10
Kinase	6.72	42	Araf, Bmp2k, Cdc42bpb, Mark1, Mark2, Mark3, Mark4, Tnik, Yes1, Adck1, Agk, Camk2b, Cask, Csnk1a1, Csnk1d, Csnk1e, Csnk2a1, Cerk, Gak, Cdk18, Cdkl5, Dgkz, Dgkb, Dgke, Ddr1, Fn3k, Gk, Gsk3b, Magi3, Mast1, Mapk3, Mapk8ip2, Map2k1, Map3k13, Pfkl, Phkg1, Prpf4b, Prkar2a, Prkg2, Pdk3, Stk39, Tnk2	1.6E-09
Receptor Clustering	3.75	5	Dlg1, Dgl2, Dlg3, Dlg4, Magi2	3.8E-03

The online STRING analysis also identifies reference publications that contain genes or proteins that show a significant overlap with the input list. Interestingly, three reviews on ASD showed significant overlap of cited proteins and SIPs identified in the study: review of ionotropic glutamate receptors in ASD and FXS (Uzunova et al., 2014) found that 20 of the 65 proteins listed overlapped with SIPs identified in the study (FDR = 8.35e-11), and a review on monogenic mouse models of ASD (Hulbert and Jiang, 2016) reported 16 of 39 genes mentioned were found in our proteomic analysis (FDR = 9.88e-10). Together, these studies point to the possibility that the finding of rare mutations in SERT in studies of subjects with ASD may reveal other protein complexes that do not directly engage SERT, but can be impacted by SERT mutations, that also drives risk for ASD. Additionally, considering genes that are associated with structural connectivity of neurons (Lin et al., 2016), 16 of 73 genes mentioned overlap with proteins identified in our study (FDR = 4.45e-07). This finding reinforces the idea that although several ASD related proteins were found to interact with SERT, an *in vivo* impact may well extend beyond consideration of circuit level perturbations driven by serotonergic dysfunction.

Finally, input of SIPs into Enrichr (Chen et al., 2013; Kuleshov et al., 2016)^1^ reveals significant overlap of genes associated with autism (*P*-value = 0.02322) based on protein-protein interaction networks that connect genes from the Online Mendelian Inheritance in Man (OMIM), an online catalog of human genes and genetic disorders. In addition, 42/461 proteins from our proteomic analysis overlap at levels above chance with proteins in the 1089 genes in the Simons Foundation Autism Research Initiative (SFARI) ASD-associated gene list^2^ (Fisher exact test *P* < 0.001).

## Discussion

SERT is a dynamically regulated protein that relies on a growing list of SIPs to traffic, stabilize and functionally regulate the transporter. Past studies have identified several SIPs that regulate SERT function. However, the identification of large molecular complexes that dictate and modulate SERT function and that are impacted by functional mutations is in its infancy. In our analysis of SIPs, 21 previously described proteins, such as CaMKII (Steinkellner et al., 2015), FLOT1 (Reisinger et al., 2018), PP2Ac (Bauman et al., 2000), and NLGN2 (Ye et al., 2016) were identified (^∗^proteins in Supplementary Table 1). Additionally, a number of novel interacting proteins, many of which have been linked to ASD (41 SAFARI genes), such as septin proteins (*Sept*) (Harper et al., 2012; Hiroi et al., 2012), DLG (*Dlg*) (Li et al., 2014; Coba et al., 2018), FMR1 (*Fmr1*) (Belmonte and Bourgeron, 2006), Neuroligin-3 (NLGN3; *Nlgn3*) (Singh and Eroglu, 2013) and SH3 and multiple ankyrin repeat domains protein 3 (SHANK3; *Shank3*) (Moessner et al., 2007) were also identified as SIPs in our efforts. The goal of this study was to illuminate networks of proteins and pathways altered by the SERT Ala56 variant, whose further analysis may help delineate serotonergic connections to disease states. Clearly, additional studies are needed to validate the importance of SIPs nominated and to define how the Gly56Ala substitution alters SERT associations.

### Previously Described SIPs

As noted above, ours studies identified a number of proteins previously reported to interact with SERT. For example, our lab originally identified the catalytic “C” subunit of the PP2A holoenzyme, PP2Ac, as a SIP (Bauman et al., 2000). PP2Ac is accompanied by an “A” scaffolding subunit and a regulatory “B” subunit (Kremmer et al., 2015). Interestingly, whereas PP2Ac exhibited a decreased interaction with SERT Ala56, the regulatory subunit B (*Ppp2r2C*) exhibited an increased interaction. Possibly, the conformation stabilized by the Gly56Ala substitution may mimic a state whereby PP2Ac is displaced from the transporter as part of a regulatory cycle. As the PP2A heterotrimer is a Ser/Thr protein phosphatase and has already been demonstrated to limit SERT phosphorylation (Bauman et al., 2000), a decreased interaction of SERT Ala56 with PP2Ac may contribute to the hyperphosphorylation of SERT Ala56 observed *in vivo* (Veenstra-VanderWeele et al., 2012). To date, the structural domain of SERT supporting PP2A interactions has yet to be defined, though the impact of the Ala56 variant reported here suggests that the N-terminus may contribute. Studies using WT and mutant N-terminal peptides as interactors with PP2A subunits are needed to define whether the changes noted are direct or indirect.

Another previously identified SIP, one that showed increased interaction with SERT Ala56 compared to WT SERT, is Sec23A/24C. Sec23A/24C, PDZ domain-containing proteins, are members of coat protein complex II (COPII) that bind to the C-terminus of SERT at amino acids Arg607-Iso608 (Chanrion et al., 2007; Sucic et al., 2011) and have been reported to mediate SERT export from the endoplasmic reticulum (ER) to the membrane surface (Sucic et al., 2011). The Ala56 variant does not affect SERT steady-state surface expression in transfected cells (Prasad et al., 2009), and thus no effort has been expended as of yet to assess if changes in transporter protein synthesis, ER export or membrane trafficking dynamics are impacted by the SERT Ala56 variant. There may be cause to revisit this idea given that the DAT Cys615 coding variant associated with ADHD has been found to undergo a different mode of membrane recycling, once transferred to the cell surface (Sakrikar et al., 2012). It should also be noted that SERT surface levels from SERT Ala56 KI mice have yet to be determined, and could show differences as compared to *in vitro* studies. Such a phenomenon has been demonstrated with another ASD-associated transporter coding variant, DAT Val559 (Gowrishankar et al., 2018), where surface expression is not altered in transfected cells, but is impacted *in vivo* due to an the ability of mutant transporter-mediated dopamine efflux to activate presynaptic D2-type dopamine receptors that regulate DAT trafficking.

Another previously identified SIP is a member of the 14-3-3 family, which are apdaptors that stabilize protein-protein interactions (Aitken et al., 2004). Specifically, 14-3-3τ has been reported to interact with the N-terminus of SERT and to lead to decreased SERT Vmax, potentially through a PKC-dependent pathway (Haase et al., 2001). However, there are multiple isoforms of 14-3-3 and two other 14-3-3 isoforms (α/β; *Ywhab* and γ; *Ywhag*) were shown to have increased interaction with WT SERT, whereas 14-3-3η (*Ywhah*) showed increased interaction with SERT Ala56. These findings, if confirmed, may indicate the use of distinct 14-3-3 isoforms in different cellular compartments of 5-HT neurons (i.e., soma, dendrites, axons). Further studies will be needed to tease apart the complexity of the interactions of 14-3-3 isoforms with SERT, each of which may play different, but important, roles in regulating SERT activity.

We have previously shown that NLGN2 (*Nlgn2*) forms a functional interaction with SERT in somatodendritic compartments, specifically within the midbrain (Ye et al., 2016), most likely at sites that support somatodendritic GABAergic synapses. Interestingly *Nlgn*2 KO mice exhibit social deficits that are mimicked in SERT Ala56 mice (Ye et al., 2016), suggesting that NLGN2 may exhibit some of its perturbations of social behavior through a SERT and 5-HT-dependent mechanism. We have speculated that SERT, 5-HT_1__A_ receptors, NLGN2, and GABA receptors may form a complex to spatially co-organize inhibitory control mechanisms that regulate 5-HT neuron excitability (Ye et al., 2016), which is diminished in the SERT Ala56 mouse (Veenstra-VanderWeele et al., 2012). Studies of GABA receptor agonist stimulation of 5-HT neurons from WT and SERT Ala56 mice are needed to test this idea, and if the model is corroborated, would add an additional dimension to the suggested contribution of an excitation/inhibition imbalance in ASD (Yizhar et al., 2011; Nelson and Valakh, 2015; Golden et al., 2018), and bridge the latter model to evidence of altered serotonergic signaling in ASD.

Previous studies identified CaMKIIβ and CaMKIIα as SIPs with CaMKIIα found to play in SERT-mediated, D-amphetamine-induced 5-HT efflux (Steinkellner et al., 2015). This is of interest considering that the Ala56 substitution was shown to blunt SERT-mediated 5-HT efflux in response to the D-amphetamine derivative, D-fenfluramine (Quinlan et al., 2019). However, it is important to note that CaMKIIβ was only found to have an increased interaction with WT SERT compared to SERT Ala56 based on normalized precursor intensity with no difference ion intensity-based quantitation, reinforcing the importance that these findings be verified by other methods. Also of note, CaMKIIα was identified in our proteomic analysis, but was eliminated due to the high levels of interaction detected in the SERT KO sample.

### Novel SIPs and Networks

In our project, while we provide evidence that multiple known SIPs were re-identified in our efforts and demonstrated to have SERT specificity, rather than validate additional interactors noted, we chose to use our data to nominate coherent networks where these molecules and others might serve as a starting point for future investigations.

One novel family of proteins found to show increased interaction with SERT Ala56 compared to WT SERT is the septin family. Septins are cytoskeletal GTPases that participate in vesicle trafficking and compartmentalization of the plasma membrane. Some studies suggest that septins may regulate exocytosis (Tokhtaeva et al., 2015). Interestingly, Sept5 interacts with Sept11 (Bläser et al., 2010), which was also identified as a SIP, and Sept5 (also referred to as CDCrel-1) which has been shown to bind to syntaxin 1A and inhibit exocytosis (Beites et al., 1999). Syntaxin 1A has been shown to form a functional complex with SERT (Quick, 2002), so potentially these findings may suggest the presence of a Sep5:Sep11: syntaxin 1A: SERT macromolecular complex, possibly localized near synapses where syntaxin 1A is enriched due its role in synaptic vesicle exocytosis, though this assumption requires direct evaluation. As to the broader relevance of this work, we note that Sept5 has been implicated in a number of diseases, including ASD (Harper et al., 2012; Hiroi et al., 2012).

Haase et al. (2017) found in their proteomic analysis of SIPs an enrichment in synaptic vesicle proteins. We identified two vesicular proteins, synaptophysin (*Syp*) and synaptotagmin-11 (*Syt11*) in the list of proteins that have increased interaction with SERT Ala56 vs. WT SERT. However, synaptic vesicles proteins were not found enriched in our specific network analysis, though it is well known that vesicle protein complexes are dynamic interactors and some components may simply not have been assembled in the extracts analyzed. Nonetheless, it seems reasonable to speculate that associations of a more active SERT Ala56 might enhance proximity of the transporter to synaptic vesicles as a means of enhancing the repacking of 5-HT. In this regard, a close association of DAT with the vesicle protein synaptogyrin-3 protein has been described where the complex has been suggested to facilitate DAT-dependent refilling of DA synaptic vesicles (Egana et al., 2009).

Another novel interesting SIP shown to interact to a higher extent with SERT Ala56 is the fragile X mental retardation 1 protein (FMR1; *Fmr1*), as well as the fragile X-related protein (*Fxr1*). Fragile X syndrome (FXS) is a single gene disorder that results in the silencing of *Fmr1*, and has considerable overlap in symptomology with ASD (Belmonte and Bourgeron, 2006) and has been linked to 5-HT dysregulation (Hanson and Hagerman, 2015). Treatment with the SSRI sertraline has been found to be beneficial for some patients with FXS. In *Fmr1* KO mice, SERT mRNA is reduced in thalamic nuclei compared to WT mice during postnatal development (Uutela et al., 2014), providing further support of FMR1 connection to SERT. FMR1 proteins are known to be RNA binding proteins that tightly regulate the translation of proteins at an active synapse (Dockendorff and Labrador, 2019), such as the metabotropic glutamate receptor mGlur5 (Bear et al., 2004). Since the function of FMR1/FXR1 is most often considered in relation to post-synaptic compartments where translational control of protein expression occurs, these findings may reflect assembly with somatodendritic transporters. Also of note, FMR1/FXR1 proteins have been shown to interact and regulate membrane protein function, independent of mRNA translation (Deng et al., 2013). Future experiments are necessary to delineate if the SERT:FMR1/FXR1 complex is maintained at the membrane or is involved in SERT mRNA translation to illuminate the role of this interaction.

In support of findings that regulation of SERT phosphorylation is impacted by the Gly56Ala substitution (Veenstra-VanderWeele et al., 2012), several serine/threonine kinases were identified to demonstrate a decreased association with SERT Ala56, including Mitogen-activated protein kinase 3 (*Mapk3*), also known as the extracellular signal-regulated kinase 1 (ERK-1). Not much is known about ERK-1 with respect to regulation of SERT (see Bermingham and Blakely, 2016 for a compressive review), except for one study that reported that inhibition of ERK-1 prevented an estradiol-induced decrease in 5-HT clearance in the rat hippocampus (Benmansour et al., 2014). By comparison, a role of ERK-1 in regulating DAT activity, surface expression, and N-terminal phosphorylation has been amply demonstrated (Moron et al., 2003; Bolan et al., 2007; Foster et al., 2012; Owens et al., 2012). Possibly, ERK-1 may contribute to SERT regulation by stimuli not yet identified. Given that SERT is regulated by other MAPK family members, specifically p38α MAPK, further studies are warranted to validate and determine the functional significance of this potential association.

Interestingly, a significant number of proteins reported in a KEGG pathway ascribed to amphetamine addiction were also identified to be less associated with SERT Ala56 compared to WT SERT. Our finding of a decreased effect of D-fenfluramine (a SERT-specific amphetamine) on 5-HT efflux in cells expressing SERT Ala56 vs. WT SERT (Quinlan et al., 2019) may relate to an inability of proteins in this network to properly modulate transporter conformations that support SERT-mediated 5-HT efflux.

The Arp2/3 protein complex was identified as an annotation cluster in our DAVID analysis of proteins with decreased interaction with SERT Ala56 compared to WT SERT (Table 2). The Arp2/3 protein complex regulates and binds to an intricate, branched actin network that plays an important role in the maturation of dendritic spines (Korobova and Kvitkina, 2010), especially during development (Chou and Wang, 2016). Disruptions in actin cytoskeleton networks may be involved in some of the mechanisms purported to contribute to neuropsychiatric disorders (Yan et al., 2016). Specifically, spine morphology has been found to be disrupted in ASD (Martínez-Cerdeño, 2017) and schizophrenia (Datta et al., 2017). Given the presence of SERT in the somatodendritic compartments of dorsal raphe 5-HT neurons, and our own work indicating a physical association with somatodendritic NLGN2 that is known to interact with postsynaptic GABA receptors (Ye et al., 2016), the Arp3/3 protein complex is worth further study as a potential determinant of SERT somatodendritic localization or trafficking. Should an Arp2/3 complex with SERT be confirmed, the altered association observed with SERT Ala56 may contribute to changes in SERT activity near somatodendritic 5-HT_1A_ receptors which could impact inhibitory feedback control of 5-HT neuron excitability. This could potentially explain the decreased basal firing rates of 5-HT neurons observed in SERT Ala56 mice (Veenstra-VanderWeele et al., 2012).

The cGMP-dependent protein kinase 2 (PKGII; *Prkg2*) was also identified to be a SIP and to be reduced in SERT Ala56 complexes. Interestingly, past studies have shown that PKGII does not interact with SERT due to the myristoylation domain that anchors PKGII to the membrane (Zhang and Rudnick, 2011) and our studies demonstrated colocalization of SERT with PKGI but not PKGII in rat serotonergic neuroblastoma cells (Steiner et al., 2009). However, both the latter studies were performed *in vitro* and it is possible that *in vivo*, SERT may localize to different lipid raft domains that allow SERT to engage in PKGII-dependent regulation. In this regard, we found PKGII levels to be very low in RN46A cells, whereas mouse midbrain expressed significant quantities of the kinase. If supported by future studies, the reduced interaction seen by PKGII with SERT Ala56 may relate to the inability of SERT Ala56 to be regulated by the PKG activator 8BrcGMP (Prasad et al., 2005; Sutcliffe et al., 2005).

The very C-terminal end of SERT contains a conserved, non-classical PDZ binding motif (NAV; amino acids 628–630), and thus it is not a surprise that a number PDZ domain binding proteins are found to interact with SERT, including nNOS, protein-1 that interacts with C-kinase (PICK1), and the channel interacting PDZ protein (Chanrion et al., 2007). Proteins within the PDZ domain network represent scaffolding proteins that regulate multiprotein complexes within the plasma membrane of both presynaptic active and postsynaptic density zones (Garner et al., 2000; Nourry et al., 2003). PDZ domain is also referred to as the *Drosophila* disks-large (DLG; *Dlg*) homology domain as genetic disruptions of these proteins in *Drosophila* have been found to cause significant changes to the morphology of synapses (Budnik, 1996). Interestingly, a number of variants of the Dlg and Dlg-associated protein (*Dlgap*) have been found in ASD patients (Li et al., 2014). Genetic elimination of *Dlgap1* in mice decreases sociability (Coba et al., 2018). Considering 5-HT plays a critical role in modulating social behavior (Muller et al., 2016; Walsh et al., 2018), perhaps some of the behavioral effects of Dlgap mutations involves altered SERT function.

Often, PDZ domains are found in combination with other interaction domains, including SH3 (Nourry et al., 2003), features of another network shown to be enriched in our SIP analysis. There is only one SH3 domain (PXXP) (Kurochkina and Guha, 2013) located within the C-terminus of SERT, amino acids 614–617 (PETP). Interestingly, Thr616 has been shown to support SERT phosphorylation *in vitro* by p38α MAPK (Sørensen et al., 2014). The role of this SH3 domain is currently unknown, however, could function as a scaffold for protein binding. Such a role has been suggested for the SH3 domain located in the DAT N-terminus, which also contains a MAPK phosphorylation site (Thr53) (Vaughan and Foster, 2013) that our lab has shown to be phosphorylated in response to D2 autoreceptor activation (Gowrishankar et al., 2018).

One protein identified as affected by the Ala56 mutation that contains both an SH3 domain and PDZ domain is the membrane scaffolding protein SHANK3 (*Shank3*), another gene where mutations have been associated with ASD (Moessner et al., 2007). SHANK3 is expressed post-synaptically in excitatory synapses and binds with neuroligins (another protein associated with ASD (Singh and Eroglu, 2013) and found on this list), and accumulating evidence suggests that ASD is in part due to dysfunction of glutamatergic synapses (Arons et al., 2012; Rojas, 2014). A number of other proteins in this list are found at excitatory synapses including a glutamate receptor (*Gria1,2,3,4*), glutamate receptor-interacting protein 1 (*Grip1*), NMDA receptor 2 (*Grin2a*) and a glutamate transporter (*Slc1a3*) isoform (Supplementary Table 1) 5-HT is a modulatory molecule that can form tripartite synapses with glutamatergic neurons (Belmer et al., 2017). The presence of SERT in somatodendritic compartments where glutamate synapses are formed on 5-HT neurons may better explain these findings since the extracts used for proteomic studies were taken from the midbrain where 5-HT neuron cell nodes are localized (Jacobs and Azmitia, 1992). In this regard, the Amara lab has implicated SLC1A3 glutamate transporters in D-amphetamine action on dopamine neurons through a somatodendritic site of action (Underhill et al., 2014).

Several SIPs associated with synapse and membrane localization were identified, which is no surprise considering SERT functions at the synapse. However, how SERT in different activity states compartmentalizes into different membrane subdomains is still not well understood. Of interest, a peptide targeted against the C-terminus of SERT, disrupting SIPs (Chanrion et al., 2007), increases SERT lateral mobility within the membrane, an effect mimicked by activation of p38 MAPK and PKG (Chang et al., 2012). We have proposed that increased mobility following activation of these kinases is due to an untethering of phosphorylated SERT from scaffolding proteins, leading to both an increase in SERT motility (Chang et al., 2012) and changes in SERT conformation that affords higher transport activity. One hypothesis that emerges from this observation is that SERT Ala56 may exhibit increased basal membrane lateral mobility as a result of a reduction in macromolecular SIP complexes whose normal function is to stabilize SERT in cell surface microdomains. This idea may also explain why we observed a greater number of SIPs whose associations were negatively impacted by the SERT Ala56 mutation, as compared to the number of SIPs whose interactions were enhanced.

Altogether, our study suggests that multiple protein complexes sustain normal SERT activity and regulation, and that the SERT associations of these SIPs are sensitive to a functional human coding variant *in vivo*. This conclusion, if further validated, is striking given the modest nature of the Gly to Ala substitution (one methyl group change). Already, we know that aspects of SERT conformation, 5-HT transport activity, phosphorylation and contribution to 5-HT clearance in the CNS are impacted, and it seems highly unlikely that the Ala56 substitution, on its own, can drive all of these changes. In that context, we suggest that the molecules and networks we nominate as determinants of SERT localization and function may harbor targets that can be captured for the development of therapeutics targeted to disorders linked to serotonergic dysfunction, including ASD. In this regard, we recently presented evidence that an indirect approach to the modulation of SERT function via p38α MAPK inhibition can normalize ASD-like physiological and behavioral alterations seen in the SERT Ala56 mouse (Robson et al., 2018).

## Materials and Methods

### Animal Usage

All experiments conducted using animal subjects were conducted according to the National Institutes of Health Guide for the Care and Use of Laboratory Animals. All experiments involving animal subjects were conducted as pre-approved by the Vanderbilt University and Florida Atlantic University Institutional Animal Care and Use Committees. SERT Ala56 and WT littermate males (129/sv background) 8–12 weeks old generated from heterozygous breeding while SERT KO mice on a C57BL/6J background were breed from homozygous breeding. Mouse genotyping was conducted as previously described (Veenstra-VanderWeele et al., 2012).

### Antibodies

SERT: guinea pig anti-5-HTT (#HTT-GP-Af1400, Frontier Institute, Japan; 1:2,000 for western blots); SERT anti-serum #48 (for co-immunoprecipitation; Qian et al., 1995); HRP-labeled β-actin antibody (Sigma-Aldrich, St. Louis, MO, United States; 1:10,000 for western blots).

### Serotonin Transporter Co-immunoprecipitation and Western Blot

To increase SERT pull down for proteomic analysis, we first optimized our immunoprecipitation conditions, considering multiple commercial and lab-generated anti-SERT antibodies, bead coupled versus uncoupled free SERT antibody, whole tissue versus synaptosomes as a tissue source, and different detergents for sample solubilization (e.g., RIPA, *n*-Dodecyl-*b*-Maltoside (DDM), CHAPS, Triton-X, and Octyl β-D-glucopyranoside). With these considerations, we pursued methods as follows: freshly dissected midbrain tissue from 4 mice were homogenized in 10% (w/v) of 0.32 M sucrose, 10 mM HEPES, 2 mM EDTA utilizing a Teflon-glass tissue homogenizer. The resulting midbrain homogenate was centrifuged for 10 min at 800 X g and the supernatant was then subjected to 10,000 × *g* spin for 10 min. Pellets were lysed for 1 h rotating in PBS + 0.7% DDM (Thermo Fisher, Waltham, MA, United States) containing protease inhibitors (P8340, 1:100; Sigma, St. Louis, MO, United States) at 4°C. Protein lysates were centrifuged for 15 min at 16,060 × *g* to obtain soluble material. Protein concentrations were determined by the BCA method (Thermo Fisher, Waltham, MA, United States). One mg of supernatant was then added to 50 μL protein A Dynabeads (Invitrogen, Carlsbad, CA, United States) that were previously cross-linked with an anti-SERT serum #48 using dimethyl pimelimidate (DMP) (Schneider et al., 1982). Briefly, beads were washed 3X in PBS at room temp followed by incubation with #48 anti-SERT serum at 4°C for 1 h. Antibody-bound beads were then incubated with 6.5 mg/mL DMP in 0.2 M Triethylamine (TEA) buffer for 30 min at room temperature. The incubation step was repeated 3X with freshly made DMP buffer each time. Cross-linked beads were then quenched in 50 mM ethanolamine (EA) buffer for 5 min at room temp, washed 2X in 1 M glycine (pH = 3) buffer, and then 3X in PBS (10 min, room temp) and then stored at 4°C until use. Immunocomplexes were eluted by incubating beads with 2X Laemmli sample buffer at 70°C for 10 min. Eluted samples were separated by 10% SDS-PAGE, blotted to PVDF (Millipore, Billerica, MA, United States) membrane and then incubated with primary and secondary antibodies at dilutions noted above. Immunoreactive bands were identified by band visualization and quantitation by enhanced chemiluminescence (Bio-Rad Clarity ECL, Hercules, CA, United States) using an ImageQuant LAS 4000 imager (GE Healthcare Life Sciences, Chicago, IL, United States) or an Odyssey FC imager (Li-Cor Biosciences, Lincoln, NE, United States). To visualize eluted SERT complexes after separation by 10% SDS-PAGE, gels were stained utilizing a Colloidal Blue staining kit (Thermo Fisher, Waltham, MA, United States).

### Liquid Chromatography-Tandem Mass Spectrometry

Proteomic analysis was performed in the Vanderbilt Proteomics Core Facility of the Mass Spectrometry Research Center. SERT immunocomplexes eluted from the antibody-conjugated Dynabeads were first resolved for 6 cm using a 10% Novex^®^ precast gel. Protein bands were excised from the gel and cut into 1 mm^3^ pieces. Proteins were treated for 30 min with 45 mM DTT, and available Cys residues were carbamidomethylated with 100 mM iodoacetamide for 45 min. Gel pieces were further destained with 50% MeCN in 25 mM ammonium bicarbonate, and proteins were digested with trypsin (10 ng/μL) in 25 mM ammonium bicarbonate overnight at 37°C. Peptides were extracted by gel dehydration with 60% MeCN, 0.1% TFA, the extracts were dried by speed vac centrifugation, and reconstituted in 0.1% formic acid. For LC-MS/MS analysis, peptides were loaded onto a self-packed biphasic C18/SCX MudPIT column using a Helium-pressurized cell (pressure bomb). The MudPIT column consisted of 360 × 150 mm i.d. fused silica, which was fitted with a filter-end fitting (IDEX Health & Science) and packed with 5 cm of Luna SCX material (5 mm, 100 Å) followed by 4 cm of Jupiter C18 material (5 mm, 300 Å, Phenomenex). Once the sample was loaded, the MudPIT column was connected using an M-520 microfilter union (IDEX Health & Science) to an analytical column (360 mm × 100 mm i.d.), equipped with a laser-pulled emitter tip and packed with 20 cm of C18 reverse phase material (Jupiter, 3 mm beads, 300 Å, Phenomenex). Using a Dionex Ultimate 3000 nanoLC and autosampler, MudPIT analysis was performed with an 8-step salt pulse gradient (0, 100, 150, 200, 300, 500, 750, and 1000 mM ammonium acetate). Following each salt pulse, peptides were gradient-eluted from the reverse analytical column at a flow rate of 350 nL/min, and the mobile phase solvents consisted of 0.1% formic acid, 99.9% water (solvent A) and 0.1% formic acid, 99.9% acetonitrile (solvent B). For the peptides from the first 7 SCX fractions, the reverse phase gradient consisted of 2–50%B in 83 min, 50%B from 83–84 min, 50–2%B from 84–85 min, and column equilibration at 2%B from 85–95 min. For the last SCX-eluted (1 M ammonium acetate) peptide fraction, the peptides were eluted from the reverse phase analytical column using a gradient of 2–98%B in 83 min, 98%B from 83–84 min, 98–2%B from 84–85 min, and 2%B from 85–95 min. Peptides were introduced via nanoelectrospray into a Q Exactive Plus mass spectrometer (Thermo Scientific, Waltham, MA, United States), and data were collected using a data-dependent method. The instrument method included an MS1 AGC target value of 3e6, followed by up to 15 MS/MS scans of the most abundant ions detected in the preceding MS scan. The MS2 AGC target was set to 1e5, dynamic exclusion was set to 30 s, HCD collision energy was set to 28 nce, and peptide match and isotope exclusion were enabled.

### Sequest and Mascot Protein Identification

Charge state deconvolution and deisotoping were not performed. All MS/MS samples were analyzed using Mascot (Matrix Science, London, United Kingdom; version 2.3.01) and Sequest (Thermo Fisher Scientific, San Jose, CA, United States; version 1.4.1.14) by The Scripps Research Institute, Jupiter, FL, United States. Both Mascot and Sequest were set up to search the UniProt Mouse2016_08_BSA release (16918 entries^3^) assuming the digestion enzyme trypsin. Mascot was searched with a fragment ion mass tolerance of 20 ppm and a parent ion tolerance of 10.0 ppm. Sequest was searched with a fragment ion mass tolerance of 0.020 Da and a parent ion tolerance of 10.0 ppm. Carbamidomethyl of cysteine was specified in Mascot and Sequest as a fixed modification. Deamidated ion of asparagine and glutamine and oxidation of methionine were specified in Mascot and Sequest as variable modifications. Scaffold (version Scaffold_4.7.3, Proteome Software Inc., Portland, OR, United States) was used to validate MS/MS based peptide and protein identifications. Peptide identifications were accepted if they could be established at greater than 89.0% probability to achieve an FDR less than 1.0% by the Scaffold Local FDR algorithm. Protein identifications were accepted if they could be established at greater than 99.0% probability to achieve an FDR less than 1.0% and contained at least 2 identified peptides. Protein probabilities were assigned by the Protein Prophet algorithm (Nesvizhskii et al., 2003). Proteins that contained similar peptides and could not be differentiated based on MS/MS analysis alone were grouped to satisfy the principles of parsimony. A total of 1050 proteins were identified. All the raw mass spectrometry proteomics data have been deposited to the ProteomeXchange Consortium via the PRIDE partner repository with the dataset identifier PXD018695.

### Label-Free Quantification by Precursor Ion Intensity and Normalized Spectral Counts

For quantification, all three replicates for each genotype were averaged. Spectral counts or the number of peptides observed per protein have been found to correlate with protein abundance and therefore has been utilized a label-free quantification method (Wong and Cagney, 2010). Normalization of total spectral counts was utilized as described by Scaffold^4^.

Another label-free quantification method is the measurement of the precursor ion intensity of the MS1 spectra (Wong and Cagney, 2010). Scaffold Q+ (version Scaffold_4.7.3, Proteome Software Inc., Portland, OR, United States) was used to quantitate. Normalization was performed iteratively (across samples) on intensities. Medians were used for averaging. Spectra data were log-transformed, pruned of those matched to multiple proteins, and weighted by an adaptive intensity weighting algorithm. Of 22133 spectra in the experiment at the given thresholds, 5672 (26%) were included in quantitation. Differentially expressed proteins were determined by applying the Mann–Whitney Test with unadjusted significance level *p* < 0.05.

To enrich for specific protein interactions, any protein only identified in SERT KO samples or were 1.5 times greater normalized spectral counts or precursor ion intensity of SERT KO compared to either WT or SERT Ala56 samples were eliminated. Also, all ribosomal proteins were removed from the list to eliminate proteins that are involved in the translation of SERT, which was not of interest for this analysis. This narrowed the final list of SERT interacting proteins from 1050 proteins to 459 proteins. To determine differential protein interactions, the log_2_ fold change of WT/Ala56 was calculated for both spectral counts and precursor ion intensity.

### Functional Clustering Analysis of SIPs by DAVID, STRING, and Enrichr

Functional clustering was broken up into 4 lists of proteins: Increased interaction with SERT Ala56 (log_2_ ion intensity less than −0.5); Decreased interaction with SERT Ala56 (log_2_ ion intensity greater than 0.5); Similar interaction between WT SERT and SERT Ala56 (log_2_ ion intensity between −0.5 and 0.5); and all identified proteins.

The Database for Annotation, Visualization and Integrated Discovery (DAVID v.6.8^5^) is a platform that allows for the identification of biological themes and gene ontology terms from a list of genes (Huang et al., 2009). The mouse genome was selected as the population background. Functional Annotation Clustering was performed as described in Haase et al. (2017) with a few exceptions. For the list of proteins with increased interaction with SERT Ala56 medium-stringency classification stringency were utilized. For the other three lists, high stringency was used.

The Search Tool for the Retrieval of Interacting Genes/Proteins (STRING^6^) analysis was utilized to build functional networks of known and predicted interacting proteins. Interaction networks were generated using all five sources: genomic context predictions, high-throughput lab experiments, co-expression, automated text mining, and previous knowledge in databases. The protein-protein enrichment analysis is based on the number of edges detected compared to the expected number edges based on the number of nodes presented (Fisher’s exact test followed by a correction for multiple testing) (Szklarczyk et al., 2017). To visualize STRING networks we utilized the Cytoscape (Shannon et al., 2003) Omics Visualizer plug-in^7^ that is linked to the StringApp (Doncheva et al., 2019) (confidence score cut-off of 0.9) followed by application of the MCL algorithm (Enright et al., 2002) using clusterMaker2 app (Morris et al., 2011), with a minimum size of 7 proteins per cluster.

The Enricher online platform^1^ (Chen et al., 2013; Kuleshov et al., 2016) was utilized to determine gene set enrichment analysis (GSEA).

### Statistical and Graphical Analyses

Data from experiments were analyzed and graphed using Prism 7.0 (GraphPad Software, Inc., La Jolla, CA, United States). For all analyses, a *P* < 0.05 was taken to infer statistical significance. Specific details of statistical tests are given in Figure Legends.

## Data Availability Statement

The dataset generated for this study can be found in the ProteomeXchange Consortium via the PRIDE partner repository with the dataset identifier PXD018695.

## Ethics Statement

The animal study was reviewed and approved by Vanderbilt University and Florida Atlantic University Institutional Animal Care and Use Committees.

## Author Contributions

MQ, MR, RY, and KR performed the experiments. MQ, KR, KS, and RB analyzed the data. MQ and RB wrote the manuscript.

## Conflict of Interest

The authors declare that the research was conducted in the absence of any commercial or financial relationships that could be construed as a potential conflict of interest.

## Footnotes

1 http://amp.pharm.mssm.edu/Enrichr/enrich?dataset=98g6w

2 https://gene.sfari.org/tools/

3 ftp://ftp.uniprot.org/pub/databases/uniprot/previous_releases/release-2016_08/

4 https://support.proteomesoftware.com/hc/en-us/articles/115002739586-Spectrum-Count-Normalization-in-Scaffold

5 https://david.ncifcrf.gov/

6 https://string-db.org

7 https://jensenlab.org/training/omicsvisualizer/
